# A Multi‐Crosslinkable Methacrylated Alginate‐GelMa Bioink for Drop‐on‐Demand Bioprinting

**DOI:** 10.1002/mabi.70278

**Published:** 2026-10-31

**Authors:** Tommi Bui, Emine Karakaya, Jessica Faber, Jonas Röder, Jörg Teßmar, Julian Bauer, Oliver Friedrich, Silvia Budday, Aldo R. Boccaccini, Rainer Detsch

**Affiliations:** ^1^ Institute of Biomaterials Department of Materials Science and Engineering Friedrich‐Alexander‐University Erlangen‐Nürnberg Erlangen Germany; ^2^ Institute of Continuum Mechanics and Biomechanics Department of Mechanical Engineering Friedrich‐Alexander‐University Erlangen‐Nürnberg Fürth Germany; ^3^ Department For Functional Materials in Medicine and Dentistry University of Würzburg Germany; ^4^ Institute of Medical Biotechnology Department of Chemical and Biological Engineering Friedrich‐Alexander‐University Erlangen‐Nürnberg Erlangen Germany

**Keywords:** biochip, biocompatibility, biofabrication, compressive strength, fourier transform infrared spectroscopy, gel permeation chromatography, materials science, nanotechnology, photopolymer, surface modification

## Abstract

To advance biofabrication, this work presents the development of a multi‐crosslinkable bioink based on methacrylated alginate di‐aldehyde (ADAMa) combined with gelatine methacryloyl (GelMa). ADAMa was synthesized using methacrylic anhydride under specific conditions, and successful functionalization as well as changes in chain length were confirmed by NMR (Nuclear Magnetic Resonance), ATR‐FTIR (Attenuated Total Reflectance—Fourier Transform Infrared Spectroscopy), and GPC (Gel Permeation Chromatography) analyses. The resulting ADAMa–GelMa bioink can be crosslinked via several independent mechanisms, including UV‐initiated photopolymerization, enzymatic crosslinking, Schiff‐base formation, and ionic gelation with calcium ions. Our study confirms an increase in the compressive modulus with increasing GelMa content, ranging from 0.3 to 30 kPa, and in vitro biocompatibility for ADAMa–GelMa bioink with enhanced cell–material interaction for a 25:75 ADAMa–GelMa ratio. Furthermore, Drop‐on‐Demand (DoD) bioprinting performance was evaluated using filament fusion test and grid structure test analyses, along with structural fidelity assessment of free‐standing pillar arrays, including metrics such as pillar height and spacing. Multiphoton microscopy further revealed homogeneous cell distribution within printed pillars. Overall, this system provides tuneable mechanical properties, high cell viability, and strong structural fidelity, making it a promising candidate for the fabrication of complex, cell‐laden constructs in advanced biofabrication applications.

## Graphical abstract

A multi‐crosslinkable methacrylated oxidized alginate–GelMA system enables precise drop‐on‐demand bioprinting by integrating tailored polymer functionality with tunable hydrogel properties. The designed macromolecular network combines photochemical, dynamic covalent, and ionic interactions to achieve printable, cytocompatible bioinks for advanced three‐dimensional biofabrication.

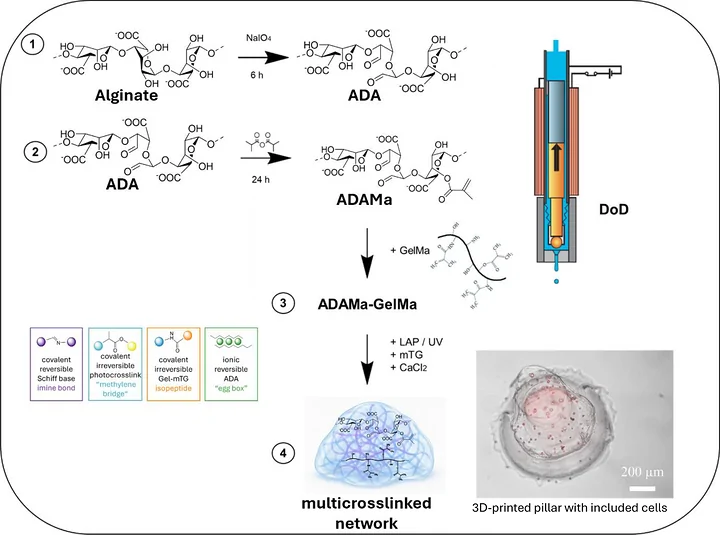

## 1 Introduction

Advanced biofabrication can be defined as the use of cutting‐edge methods to produce complex biological systems, for example for personalised and regenerative medicine. While this work focuses on biofabrication, it should be noted that additive manufacturing technologies have also become increasingly important in various clinical disciplines, including reconstructive surgery, tumour treatment, and wound healing, illustrating the versatility and translational potential of 3D printing technologies [1, 2, 3, 4, 5, 6, 7, 8, 9, 10].

An advanced method of biofabrication is the additive manufacturing process of Drop‐on‐Demand bioprinting (DoD), in which individual droplets of material are moulded and applied. Compared to, e.g., extrusion or continuous inkjet printing, cell‐laden inks can be dosed more accurately and placed droplet by droplet at a relatively fast speed and high resolution [11]. The possible drawbacks of the method are high shear stresses during printing, the difficulty of printing complex structures in the z‐axis, and the requirement for low‐viscosity bioinks [2, 12].

The central components of biofabrication are the bioinks, which require a set of properties including biocompatibility, biomimicry, biodegradability and stability in physiological environments [13]. Thus, bioink development is often seen as a bottleneck in biofabrication, where the capabilities of bioprinters cannot be fully utilised [14].

Within the field of biofabrication, printed hydrogel scaffolds have been of interest due to their high water content and physico‐mechanical properties that can match the natural extracellular matrix [15]. As an example, systems based on alginate and gelatine have been found to be potent bioinks for extrusion‐based bioprinting [16], but are conventionally not suitable for DoD bioprinting due to high viscosity [17, 18]. Alginate (Alg) and Gelatine (Gel) are biopolymers, sourced from algae and animals, that have been shown to be biocompatible and crosslinkable with different mechanisms [17, 19]. Alg can crosslink ionically with multivalent cations [20], whereas Gel can gelate thermally through triple helix formation [21]. Oxidized alginate predominantly forms alginate di‐aldehyde (ADA), which in turn can covalently bond with amino acids, such as those from Gel, through imide bonding [22]. Gelatine can further be irreversibly crosslinked by enzymatic isopeptide bonding [23]. When radically photopolymerized by functionalization with methacrylic acid, the multi‐crosslinkable inks gain an additional covalent, yet contact‐less gelation mechanism. Depending on the chemical composition of the biopolymer and the degree of functionalization, the degradability, structure, and mechanical properties could be modified [24, 25, 26]. This range of adaptability with different mechanisms of gelation can be made use of in biomaterial inks to tackle the aforementioned problems, such as the need for low viscosity during printing and a high mechanical stability thereafter. Gelatine methacryloyl (GelMa) is commonly synthesized using methacrylic anhydride (MAAh). On the other hand, Alginate di‐aldehyde methacrylate (ADAMa), which can also be modified with MAAh, is more often synthesized with 2‐aminoethyl methacrylate (AEMa) [26, 27]. The former reacts with the hydroxyl groups through esterification, while the latter reacts with carbonyl‐groups [28]. The high degradation rate and consequently low mechanical strength could be reasons for the less common esterification route [26, 29, 30]. The conjugation of the acid functions using EDC/NHS chemistry, on the other hand, might sacrifice the capability to form ionic crosslinks with calcium. While AEMa‐based ADAMa mixed with GelMa has been analyzed before to result in a wide range of coacervate formation, the combination with MAAh‐based ADAMa is yet to be discussed [30].

The aim of this study was to engineer a very low‐viscosity ADAMa–GelMa bioink incorporating photopolymer, Schiff‐base, and ionic crosslinking mechanisms to enable Drop‐on‐Demand bioprinting. The four categories of properties—chemical, physico‐mechanical, biological, and bioprinting—were selected for the purpose of determining the suitability of this bioink. This evaluation will facilitate a comparison of the bioink system with previous developments, thereby highlighting and discussing its capabilities for DoD bioprinting.

## 2 Materials and Methods

The following chemicals were used during synthesis, sample preparation, and characterization.

Alg was obtained from JRS Pharma (PH163S2, J.Rettenmaier & Söhne GmbH + Co KG, Germany). Sodium (meta)periodate (NaIO_4_, 99%), ethanol (99.8%), ethylene glycol, methacrylic anhydride (MAAh, 94%), hydrochloric acid (HCl), sodium hydroxide (NaOH) and deuterium oxide (D_2_O) were purchased from Sigma‐Aldrich (Germany). GelMa was provided by CELLINK (50% degree of functionalization, USA).

Dulbecco's phosphate‐buffered saline (DPBS, no calcium, no magnesium) was supplied by Thermo Fisher (Germany). Calcium chloride dihydrate and Lithium‐phenyl‐2,4,6‐trimethylbenzoylphosphinate (LAP, ≥95%) were bought from Sigma‐Aldrich (Germany), and microbial transglutaminase (mTG) from *streptoverticillium mobaraense* was provided by Ajinomoto Foods Europe (Germany). Glutaraldehyde and paraformaldehyde were purchased from Sigma‐Aldrich (Germany). For cell culture, DMEM (4.5 g/L glucose, Thermo Fisher, Germany) was supplemented with 1% penicillin‐streptomycin (PenStrep) obtained from Thermo Fisher (Germany) and 10% bovine calf serum (BCS) from Corning (Germany). Trypan blue was procured from Thermo Fisher (Germany), and ultra‐pure water (UPW) was produced using a Milli‐Q system (Merck, Germany).

### 2.1 Bioink Synthesis

Alg (10 g), extracted from Laminaria digitata, was partially oxidized using NaIO_4_ as described by Karakaya et al. [31]. Briefly, Alg was dispersed in 50 mL of ethanol, and NaIO_4_ was dissolved in 50 mL of UPW. Both solutions were combined and stirred for 6 h in the absence of light. The reaction was quenched by the addition of ethylene glycol, followed by further stirring for 30 min. The mixture was then decanted, and the product was dissolved in approximately 500 mL UPW. To minimize batch‐to‐batch variation, three parallel ADA syntheses were performed on the same day under identical conditions. Just prior to dialysis, the three batches were pooled. The combined solution was transferred into four dialysis membranes (SpectraPor, MWCO: 6–8 kDa, 40 mm nominal flat width, 25 mm diameter, Repligen Co., USA), each approximately 35–40 cm in length, and dialyzed against 17 L of UPW for 4 days with daily water changes. Both the synthesis and dialysis steps were carried out in complete darkness. The purified product was subsequently lyophilized (ALPHA 1–2 LDplus, Martin Christ Gefriertrocknungsanlagen GmbH, Germany), yielding solid ADA with a final yield exceeding 70%. In the second step, 2 mg of ADA lyophilizate was dissolved in 100 mL of DPBS in a darkened glass bottle. After cooling the solution to 4 °C, 33.82 mL of MAAh was added dropwise under continuous stirring in an ice bath, while maintaining light protection throughout. This volume corresponds to a tenfold molar excess of MAAh relative to all hydroxyl groups on the alginate backbone. The pH of the reaction mixture was monitored using a pH meter (FiveEasy, Mettler‐Toledo, Germany) and adjusted to pH 8 by the hourly addition of either 0.1 M NaOH or 0.1 M HCl during the first 3 h. The reaction was then allowed to proceed under continuous stirring at 4 °C overnight. After 22 h, the pH was rechecked and adjusted again, if necessary, with additional hourly measurements during the final 3 h, completing a total reaction time of 24 h. Throughout the entire process, the reaction mixture was kept at 4 °C and shielded from light.

Following the reaction, the mixture was transferred into a beaker under light‐protected conditions and allowed to rest for 5 min to enable phase separation. Unreacted MAAh and insoluble salts appeared as a visible white precipitate at the bottom, while ADAMa remained dissolved in the clear supernatant. The supernatant was carefully decanted into a dark glass bottle and diluted with 200 mL of UPW. The solution was then transferred into dialysis membranes and dialyzed against 17 L of UPW for 3 days with daily water changes, all steps conducted in the absence of light. After dialysis, further white solids (excess MAAh and insoluble salts) were observed within the dialysis tubes. To separate this from the purified product, each membrane was twisted at the appropriate location to isolate the precipitate from the rest of the solution. Only the clear supernatant was collected and pooled from all dialysis tubes (Figure 1). The resulting solution was frozen at –20 °C and subsequently lyophilized. The final ADAMa lyophilizates were stored protected from light at –20 °C until further use.

**FIGURE 1:**
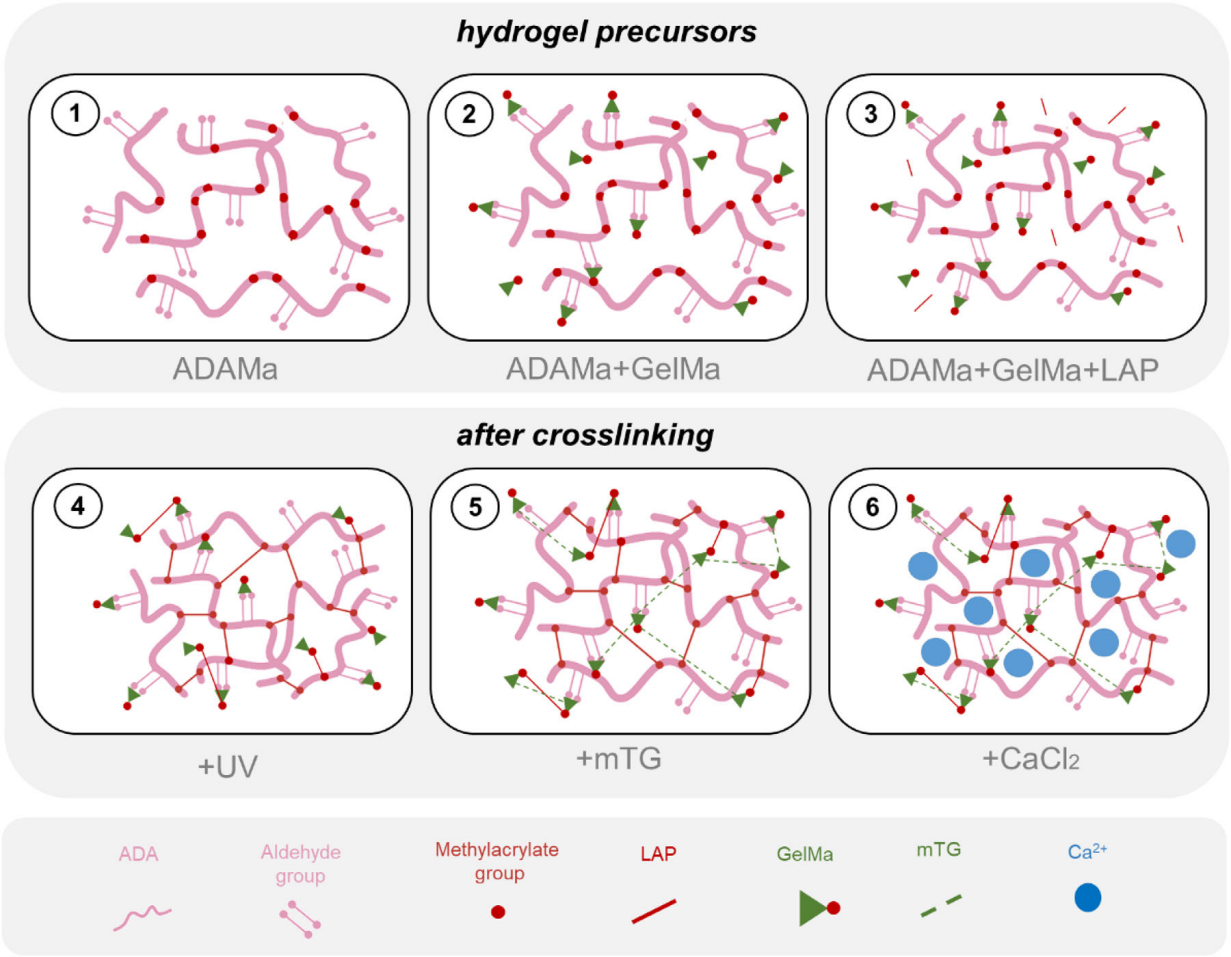
Schemata of chemical design of multi‐crosslinkable bioinks composed of alginate di‐aldehyde methacrylate and gelatine methacryloyl.

### 2.2 Preparation of Bioink Precursors

Bioink precursor solutions were prepared by mixing lyophilizates of ADAMa and GelMa individually with DPBS and 0.1‐0.3 w/v% of the water‐soluble photo‐initiator LAP. If needed, ADAMa solutions were readjusted with NaOH or HCl to a pH value of 7 while keeping the LAP concentration constant. GelMa was reconstituted in a water bath at 37°C for a minimum of 3 h to ensure complete dissolution. The precursor solutions were combined by pipetting the individual precursors in different ratios of ADAMa‐GelMa, specifically 100:0 (ADAMa100), 75:25 (ADAMa75), 50:50 (ADAMa50), 25:75 (ADAMa25) and 0:100 (GelMa).

### 2.3 Crosslinking of Hydrogel

The hydrogel precursors were photopolymerized with UV lamps at a 405 nm wavelength. UV LED Spot Curing System NSC4 (UVET, China) was used to create hydrogel films for mechanical tests, ATR‐FTIR, and cytocompatibility tests. The built‐in UV lamp from the BioX printer (Gen 3, CELLINK, USA) was used for photocrosslinking individual layers after printing. The height from the lamp to the substrate surface and the power were fixed at 3.8 cm and 30% for the former and 6 cm and 100% for the latter to achieve comparable intensities, when compared with the manufacturers’ data. After washing in DPBS, part of the photocrosslinked samples were further crosslinked by immersing in solutions that contain 0.1 M CaCl_2_ or both 0.1 M CaCl_2_ and 2 w/v% mTG for 10 min. For rheology tests, the solutions were photocrosslinked for 120 s within 2 mL centrifugation tubes and further crosslinked inside 2 mL centrifugation tubes without a washing step by pipetting the aforementioned crosslinking solutions on top and immersing for 10 min.

### 2.4 Chemical Analysis

The success of the modification and the degree of methacrylation (%methacrylation) were estimated similarly to Boddupalli and Bratlie et al. [32] using ^1^H NMR spectroscopy. For the measurements, 10 mg of ADA or ADAMa were dissolved in 0.8 mL D_2_O overnight at RT, protected from light. The samples were analyzed using a 400 MHz NMR spectrometer. Spectra were acquired at RT and 70°C with 64 scans for each sample. The %methacrylation was calculated by normalizing the integrated vinyl (I_Vinyl_: 6.1‐6.8 ppm) or methyl (I_Methyl_: 2.2–2.4 ppm) proton signals of the methacrylate groups to their respective proton numbers and relating their sum to the normalized integral of the polysaccharide backbone protons (I_Backbone_: 3.9–5.7 ppm) after subtracting the integral of the residual non‐deuterated water (4.6–4.8 ppm) according to the following equation:

(1) $$\%methacrylation=\frac{\left(\frac{I_{Vinyl}}{n_{Vinyl}}\right)}{\left(\frac{I_{Backbone}-I_{HDO}}{n_{Backbone}}\right)}or\frac{\left(\frac{I_{Methyl}}{n_{Methyl}}\right)}{\left(\frac{I_{Backbone}-I_{HDO}}{n_{Backbone}}\right)}$$

where *I_Vinyl_* and *I_Methyl_* are the integrals of the methacrylate vinyl and methyl protons, with *n_Vinyl_* = 2 and *n_Methyl_* = 3. *I_Backbone_* represents the integral of the alginate ring protons and *n_Backbone_* = 4 depicts the number of non‐exchangeable backbone protons per monomer unit.

Gel permeation chromatography (GPC) analysis of alginate and its modified derivatives was conducted using a Malvern system (Herrenberg, Germany) equipped with two A6000 M mixed‐bed columns and triple detection (refractive index, multi‐angle light scattering detector and viscometer). Calibration was performed with one commercial PEG standard of 65 kDa (Malvern, Germany). The eluent comprised deionized water containing 0.2 g L^−1^ NaN_3_ and 8.5 g L^−1^ NaNO_3_. Samples were dissolved in the eluent at room temperature for six hours prior to measurement, which was carried out at 35 °C with a flow rate of 0.7 mL min^−^^1^. Each measurement was repeated a minimum of three times, and the resulting data were processed using OmniSEC 5.12 software (Malvern, Germany).

FTIR was used to qualitatively confirm the success of the ADAMa synthesis. For this purpose, ADAMa was analysed before and after crosslinking with LAP and UV light and compared to ADA. ATR‐FTIR spectroscopy (IRAffinity‐1S, Shimadzu, Japan) was used on powders and lyophilized polymers to analyze the chemical modification of the materials. 40 scans were done with a resolution of 4 cm^−1^ and a range of 4000–400 cm^−1^. The values were each normalized between 0 and 1 and shifted vertically for better comparability.

To quantify the degree of oxidation (%DO) in ADA, a titration‐based method was applied as described by Karakaya et al.  [24]. For this purpose, 0.1 g of lyophilized ADA was dissolved in 20 mL of 0.25 N hydroxylamine hydrochloride solution. The pH of the solution was adjusted to 4.0 using 0.1 M HCl or NaOH and stirred at room temperature for 2 h to allow the aldehyde groups to react with hydroxylamine and form oximes, which released protons. These were quantified by back‐titration using 0.1 M NaOH to a pH value of 4. The volume of base consumed was then used to calculate the %DO using the following equation (2):

(2) $$\begin{array}{ccc} & & degreeofoxidation\\ & & =\;\%DO=\left(\frac{M_{NaOH}\cdot \left(V_{sample}-V_{control}\right)}{\left(\frac{m_{ADA}}{M_{Alg}}\right)}\right)\cdot \frac{1}{2}\cdot 100 \end{array}$$

where:

- *M_NaOH_* = *molarity* *of* *NaOH* (0.1 *mol*/*L*)
- *V_sample_* = *volume* *of* *NaOH* *used* *for* *ADA* *sample* (*in* *L*)
- *V_control_* = *volume* *of* *NaOH* *used* *for* *blank* *sample* (*in* *L*)
- *m_ADA_* = *mass* *of* *ADA* *sample* (0.1 *g*)
- *M_Alg_* = *molecular* *weight* *of* *one* *alginate* *monomer* *unit* (*approx*. 198 *g*/*mol*)

### 2.5 Physical Characterization and Mechanical Analysis

The optical and rheological properties (see supplements) of the precursors were identified to determine non‐chemical parameters that can affect the gelation. Once characterized, compression tests were performed to evaluate the effect of crosslinking.

Rheological measurements were performed using a rotational rheometer (HR30, TA Instruments, USA) equipped with a stainless steel plate‐plate geometry (Plate SST, diameter of 40 mm) and a gap of 500 µm. Crosslinked samples were pipetted onto the measurement plate without supernatant and trimmed at a trim offset, 25 µm above the measurement gap. Each measurement was conducted once to determine minimal lyophilizate content for crosslinking and printing. Frequency sweeps were performed at 37°C ranging from 0.01–100 s^−1^ with an amplitude of 1% strain.

Films (n = 4) with 4 w/v% lyophilizate content were fabricated by pipetting 36 µL into the chambers of a silicone mould (5 mm diameter x 2 mm height) placed within a glass petri dish, followed by crosslinking with a 405 nm LED for 120 s and 0.1 M CaCl_2_ alone or with 2 w/v% mTG for 10 min. After crosslinking, samples were stored at 4°C in DPBS overnight before being tested the following day. Using a MicroTester LT (CellScale, Canada), samples were put in a water bath at 37°C and compressed with a metal beam. The testing protocol consists of a cyclic compression test with four loading cycles with a strain rate of 0.005 s^−1^ up to a maximum strain of 10%, a holding time of 5 s after loading, a recovery time of 20 s, and a resting time of 5 s before starting the subsequent cycle. The first loading cycle was discounted as pre‐loading. Assuming that the samples are ideally cylindrical and defining the stiffness as the slope between force and displacement between 2% and 5%, the effective compressive modulus was determined by dividing the force by the cross‐sectional area and multiplying this by the initial sample height divided by the change in sample height. The diameter and height were measured with the optical system of the testing device.

For morphology studies, SEM (scanning electron microscope, Auriga CrossBeam Unit, Carl Zeiss, Germany) and Two‐Photon Microscopy (Trim‐Scope II, LaVision BioTec, Bielefeld, Germany) were used. Samples were fixed with 0.1% glutaraldehyde and 2% paraformaldehyde overnight and after that with 0.3% glutaraldehyde and 3% paraformaldehyde for 1 h. SEM samples were critical point dried (EP CPD300, Leica, Germany) after dehydrating in ethanol solutions (30, 50, 70, 80, 90, 95, 99.5%) for 25 min each.

### 2.6 In Vitro Biocompatibility

In order to evaluate the cytocompatibility of the samples, two‐dimensional (2D) cell seeding on hydrogel films (n = 3) was performed. These were created by crosslinking a sterile‐filtered precursor solution in a 48‐well plate, as previously described. The samples were exposed to 405 nm irradiation for 120 s and then to 0.1 M CaCl_2_ and 2 w/v% mTG solution for 10 min. After washing with PBS, the films were seeded with 0.5 mL of a concentrated 10^5^ cell/mL suspension, containing hTERT BJ1 td‐tomato‐farnesly reporter cells (provided by the Institute for Biochemistry and Molecular Medicine, Friedrich‐Alexander‐Universität, Germany), DMEM, 10% BCS and 1% PenStrep. The cell concentration of solutions was determined with a Neubauer Chamber. The samples were incubated at 37°C and 5% CO_2_ for 1 and 8 days. After the incubation time, the seeded films were washed with DPBS and again incubated with 300 µL cell medium containing 1% WST‐8 kit (Sigma‐Aldrich, Germany) for 4 h at 37°C and 5% CO_2_. The absorbance of the supernatant for each well (2 × 100 µL) was measured at a wavelength of 450 nm by a microplate reader (PHOMO, Anthos Mikrosysteme GmbH, Germany) to measure cell viability. The absolute value of absorption from the reference medium was subtracted from the data, and relative values for each day of measurement were calculated for better comparability.

### 2.7 Bioprinting

For all of the following tests, a diameter of 100 µm electromagnetic valve‐based nozzle and 37°C printing temperature were used. Photocrosslinking was done after every layer for 30 s.

First, the printing process was evaluated by printability tests (n = 4), commonly done with extrusion, using continuous strands. This needs to be done to ensure comparability with other methods. For that, the moving speed of the print head was set to 15 mm/s, and the opening and closing times of the valve were set to 1 and 25 ms, respectively. The pressure was set to 37 kPa for GelMa, 100 kPa for ADAMa, and was partly increased to 170 kPa for ADAMa concentrations above 2 w/v%. The Filament Fusion Test (FFT) shows segments with decreasing line distance (2, 1.5, 1, 0.75, and 0.5 mm). Through this test, the resolution of printed lines is evaluated. The Grid Structure Test (GST) shows a grid with a constant segment size of 2 mm. These grids with a 10 mm overall side length were printed in two layers with a 90° rotation. In the GST, pore shapes and strut sizes were evaluated. The printability was determined by the following Equation (3):

(3) $$Printability=\frac{\pi }{4}\cdot \frac{1}{Circularity}$$

The circularity was determined using *(Fiji Is Just) ImageJ* software (Version 2.9.0/1.53t).

4 w/v% hydrogel pillars were printed by increasing the distance between droplets and crosslinking for 30 s, layer‐by‐layer, while increasing the height by 0.1 µm for each step. The closing time was increased to 200 ms, while the opening time remained at 1 ms. The print head speed was also kept at 15 mm/s. Second, for bioprinting, the fibroblast reporter cells were centrifuged at 300 g for 5 min and dispersed in the corresponding amount of high‐glucose DMEM with 10% FCS and 1% PenStrep. This suspension was then mixed into a 4 w/v% ink solution with a ratio of 1:10 by pipetting. Hydrogel pillars with embedded cells were printed with a concentration of 10^6^ cells/mL for GelMa and ADAMa.

### 2.8 Multiphoton Microscopy

The bioprinted constructs were imaged using a multiphoton microscope (Trim‐Scope II, LaVision BioTec, Bielefeld, Germany) coupled with a mode‐locked femtosecond‐pulsed Ti:Sa laser (Chameleon Vision II, Coherent, Santa Clara, CA, USA; pulse frequency: 80 MHz). The Laser was focused into the sample with a 10x dry objective (Nikon CFI Plan Apochromat Lambda D 10X, Nikon Europe B.V., Amstelveen, The Netherlands). The fluorescence signal (TdTomato, hTERT BJ1 td‐tomato‐farnesly reporter cells, Institute of Biochemistry and Molecular Biology, FAU, Germany) was detected, utilising a photomultiplier tube (H 7422–40 LV 5 M, Hamamatsu Photonics, Germany), which was equipped with a bandpass filter (Chroma T‐series, Chroma Technology Corporation, Bellow Falls, VT, USA) to detect the spectral emission range of the applied fluorophore selectively. In order to obtain details of the 3D architecture of the construct and the embedded cells in the hydrogel, 3D image stacks were recorded (lateral pixel size: 1 µm, line scanning frequency: 1000 Hz, step size axial direction:

1 µm; pixel dwell time: 0.71 µs, physical voxel size: 1 × 1 × 1 µm^3^). Images were analysed and compiled via ImageJ/Fiji for 3D visualisation. The cylindrical sample was imaged lying on its radial mantle surface, to ensure a lower sample height and hence a better chance of complete optical penetration.

### 2.9 Statistics

To evaluate differences in mean values, one‐way and two‐way analysis of variance was performed using Origin 2022 (version: 9.9.0.225, OriginLab Corporation, Northampton, USA), depending on the number of variables. Multiple replicates (n) were prepared for analysis, as described in the respective sections. Data are presented as mean ± standard deviation (SD). Statistical significance was further assessed with Tukey's Honestly Significant Difference using the following levels of significance: ^*^ *p* < 0.05, ^**^ *p* < 0.01, and ^***^ *p* < 0.001.

## 3 Results and Discussion

The backbone of the bioink consists of ADAMa, which carries several reactive groups. ADA provides aldehyde groups that can react with amino‐containing components through Schiff base formation (Figure 1). In addition, the Ma‐groups introduced during methacrylation enable photocrosslinking upon UV exposure. Moreover, ADAMa retains the ability to form ionically crosslinked networks in the presence of divalent Ca^2^^+^. In the second step, GelMa is added to the ADAMa solution. This results in the formation of dynamic covalent bonds between the aldehyde groups of ADA and the amino groups of GelMA. These imine (Schiff base) linkages are known to be reversible under physiological conditions, allowing for dynamic bond breaking and reformation within the network. To enable photoinitiated crosslinking, lithium phenyl‐2,4,6‐trimethylbenzoylphosphinate (LAP) is added to the mixture. LAP activates the methacrylate groups in both ADAMa and GelMa, priming the system for subsequent photocuring under UV light. Upon UV exposure, the MA groups of both ADAMa and GelMa undergo free‐radical polymerization, forming a stable, covalently crosslinked network. This process results in both homogeneous (GelMa–GelMa and ADAMa–ADAMa) and heterogeneous (GelMa‐ADAMa) crosslinks, producing a robust construct with enhanced mechanical integrity and maintained shape fidelity. Subsequent addition of mTG further stabilizes the GelMa component of the hydrogel by catalysing the formation of covalent ε‐(γ‐glutamyl)lysine isopeptide bonds between glutamine and lysine residues in GelMa, thereby reinforcing the protein‐based network. In the final step, CaCl_2_ is introduced to induce ionic crosslinking of the alginate moieties in ADAMa. This additional network enhances the overall structural stability and form retention of the hydrogel construct.

The properties with regard to chemistry, physico‐mechanics, biocompatibility and bioprintability will be characterized and interpreted. Arguments for and against the suitability as a bioink will be described, weighed against one another and then summarized.

### 3.1 Chemical Characterization

Chemical analysis of ADA revealed a degree of oxidation of 13% in previous studies [31]. Furthermore, the methylacrylation degree was estimated using ^1^H NMR spectroscopy measured at both room temperature and 70°C (see Figure 2, A and B). The spectra showed a clear shift of the relevant signal regions from 6.25–5.5 ppm to 6.7–5.9 ppm, which improved peak separation and facilitated a more accurate integration of the signals. Comparison of the reference ADA spectrum with the ADAMa spectrum (see Figure 2,B) revealed new resonances in the ranges 6.1–6.8 ppm (vinyl region, I_Vinyl_) and 2.2–2.4 ppm (methyl region, I_Methyl_), confirming the successful conjugation of methacrylic acid to the different hydroxyl groups of alginate. The newly introduced signals in these regions were used to estimate the achieved substitution degree using Equation 1, resulting in a %methacrylation of approximately 20% (24.3% or 17.9% depending on the considered NMR signal, see Table S1). %methacrylation of alginate typically ranges from approximately 10%–30% under mild reaction conditions, whereas higher amounts of methacrylic anhydride or extended reaction times can yield degrees of substitution up to 40%–60 % [28, 33, 34]. For example, it was reported that the 55% methacrylation demonstrated that highly functionalised alginate derivatives retained the capacity to form stable, mechanically robust photocrosslinked hydrogels [35]. Therefore, a substitution degree of around 20 % indicates an efficient functionalization of the polymer, leading to a sufficient density of methacrylate groups providing sufficient photocrosslinking capability while maintaining enough flexibility and solubility.

**FIGURE 2:**
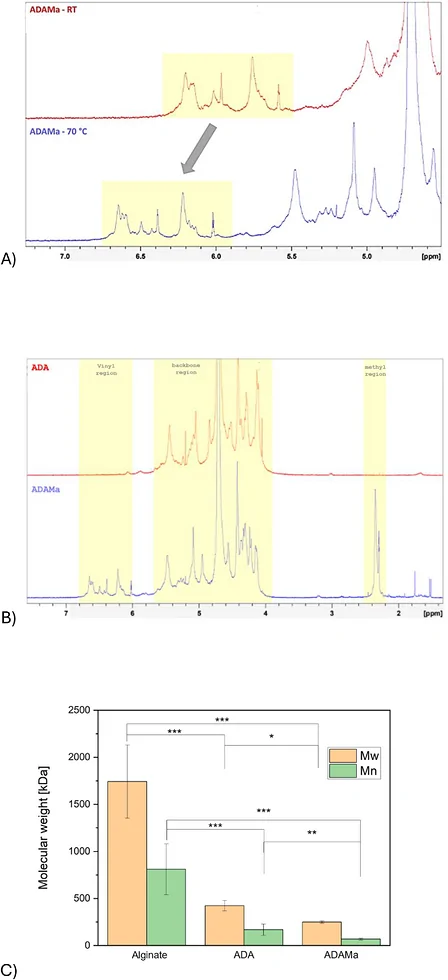
A: 1H NMR spectra of ADAMa measured at RT and 70°C showing a signal region shift from 6.25‐5.5 ppm to 6.7‐5.9 ppm (top). 1H NMR spectra of ADA and ADAMa, both measured at 70°C, depicting characteristic signals in the 6.1‐6.8 ppm (vinyl region, IVinyl), 3.9‐5.7 ppm (backbone region, IBackbone) and 2.2‐2.4 ppm (methyl region, IMethyl) used for the calculation of %methylacrylation (B). C: Weight‐average (Mw) and number‐average (Mn) molecular weights of alginate, ADA, and ADAMa derivatives used in this study, as obtained by GPC using triple calibration. Statistical significance was assessed with Tukey's Honestly Significant Difference using the following levels of significance: *p < 0.05, **p < 0.01, and ***p < 0.001.

The weight‐average molecular weight of the alginate utilized was approximately 2 MDa (Mw = 1,743 ± 388 kDa; Mn = 810 ± 269 kDa), with a dispersity (Ð) of 2.2 calculated using the performed triple calibration, which aligns with expectations for a natural polymer sourced from brown algae (see Figure 2,C). Following oxidation to generate oxidized alginate (ADA), significant degradation of the polymer occurred, reducing its molecular weight to less than 25% of the original value (Mw = 423 ± 55 kDa; Mn = 168 ± 60 kDa), with a dispersity of 2.5, as corroborated by previous studies. Subsequent functionalization with methacrylate further diminished the molecular weight by half, yielding a Mw of 249 ± 13 kDa and an Mn of 68 ± 11 kDa, accompanied by an increased dispersity of 3.7. Overall, the chemical modification of alginate results in significant degradation of the polysaccharide backbone during oxidation and methacrylation. The final modification step reduces the molecular weight by approximately 50%, potentially due to light‐induced radical crosslinking of methacrylated alginate chains, which is associated with an increase in dispersity. Hydrolytic degradation seems to be one main cause for the observed decrease, considering that esters, such as those found in ADAMa, are susceptible to hydrolysis [36], that a pH value lower than 6 leads to hydrolytic degradation of ADA [37], and that a value of around 4 was measured during and after the synthesis due to the acidic nature of MAAh. A different synthesis approach where ADA was also methacrylated with MAAh maintained a pH of 8, used ethanol, and an additional drying process to result in an ADAMa with M_w_ of 125 kDa and an increase in molecular weight instead [26]. The difference to our tested M_w_ of 254 kDa could also be related to a difference in molecular weight in the sources of alginate. An increase in molecular weight, on the other hand, can be attributed to photopolymerization. For the purpose of DoD bioprinting, a controlled decrease in M_w_ is desired due to the associated decrease in viscosity during printing and in mechanical strength after printing. Lower‐molecular‐weight alginates have also been reported to exhibit faster degradation kinetics and to facilitate improved cell infiltration, which can be advantageous for tissue engineering applications [26]. This may be achieved through an optimized synthesis method and closely monitoring reaction conditions such as pH and light exposure.

The ATR‐FTIR spectra show a characteristic peak at 1720 cm^−1^ consistent with ester bond formation that results from methacrylic acid bonding to hydroxyl groups (see Figure 3). This suggests the successful synthesis of ADAMa [26]. With prolonged UV light exposure of 120 s, both ADAMa and GelMa indicate ‐CH stretching with characteristic peaks at 2920 and 2850 cm^−1^ [38]. The appearance of these additional vibrational modes is evidence for the crosslinking reaction. The time of occurrence for these peaks after a crosslinking time of 120 s at 405 nm with LAP as photoinitiator is shorter than otherwise published, where different ADAMa systems were crosslinked for 6 min and higher at 485 nm for further characterization [26, 39]. In DoD of water‐based inks, the shortest possible crosslinking time is desired due to the fast evaporation time of small volume droplets [40]. Light‐activated bioprinting, on the other hand, requires a moderate to low concentration of radicals due to considerations regarding biotoxicity [41]. Hence, the degree of functionalization, photoinitiator concentration, light wavelength, and irradiation time have to be modulated appropriately. In previous experiments, AlMa was synthesized similarly to ADAMa. Both AlMa and ADAMa show the same carbonyl stretching bond without noticeable peak shift or broadening, indicating comparable methacrylate ester formation (see Figure S1).

**FIGURE 3:**
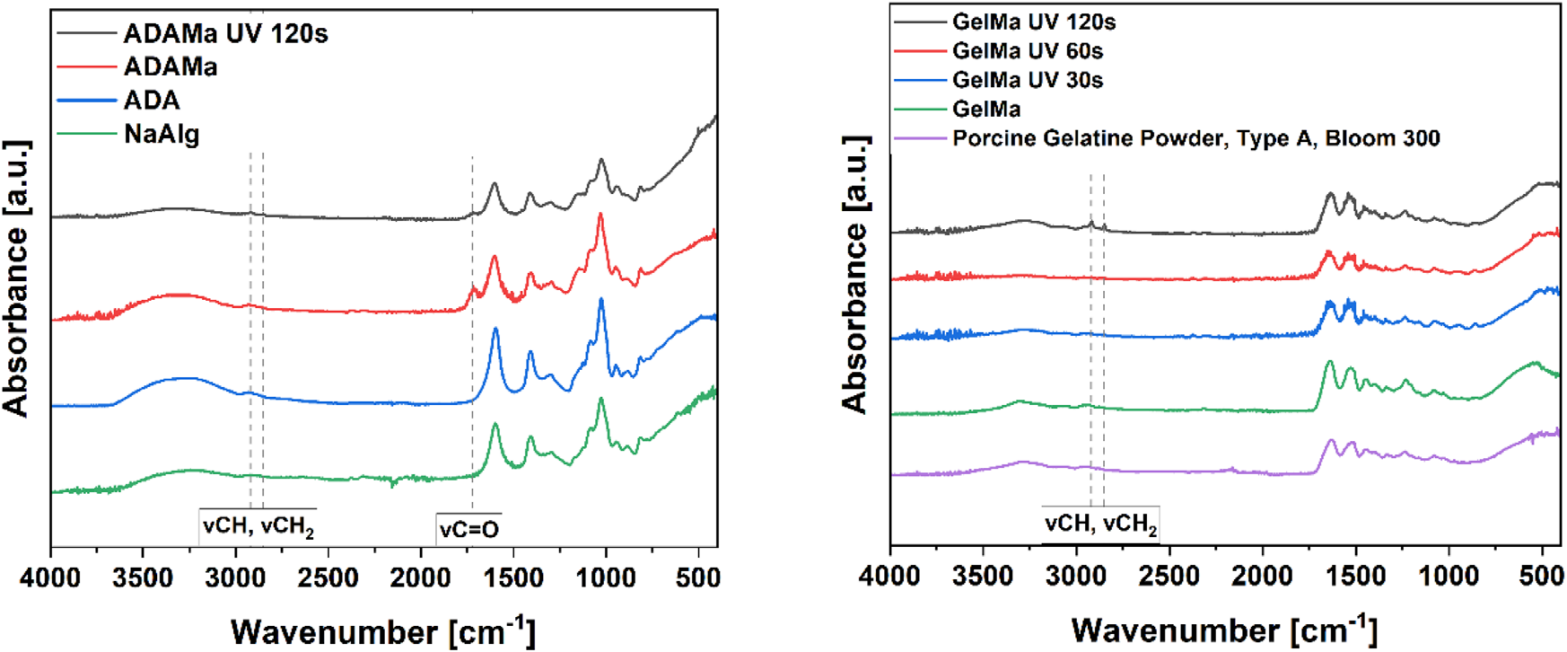
ATR‐FTIR spectra for alginate, gelatine powder, and freeze‐dried derivatives without and with exposure to LED light at 405 nm for different lengths of time.

With NMR, GPC, and ATR‐FTIR, the chemical structure of the components was identified and can serve as reference points for the following. The results indicate the right direction for DoD bioprinting—a shorter crosslinking time at moderate wavelength for successful photocrosslinking of low‐viscosity bioink at the desired time and place. Given the complex chemical nature of methacrylated alginate and the extensive dialysis process carried out following synthesis, unreacted reagents and low‐molecular‐weight by‐products should be removed. Nevertheless, a more detailed structural analysis using complementary analytical approaches would be beneficial for future studies.

### 3.2 Physical and Mechanical Characterization

Coacervate formation was observed for ADAMa‐GelMa mixtures with an ADAMa ratio of 50% and higher (see Figure S2). This window of miscibility is bigger compared to AEMA‐modified ADAMa [30]. As seen in the UV–vis spectra, coacervate formation hinders the transmittance of UV radiation. Structural homogeneity cannot be guaranteed due to the non‐stationary de‐mixed phase, but also due to differences in penetration depth for photopolymerization. As such, ADAMa25 is characterized as the optimal mixing ratio for DoD bioprinting.

Rheological flow sweep measurements show typical shear thinning behavior (data not shown) and gel‐like behavior (G′>G″) for photocrosslinked samples with a polymer concentration above 0.6 w/v% (see Figure 4). On the other hand, samples multi‐crosslinked with UV light exposure, CaCl_2,_ and mTG showed shear thinning and behaved like a gel down to 0.6 w/v%. A thermal ramp measurement also showed thermal gelation for ADAMa25 at the same concentration (see Figure S3). Thus, a critical gel concentration (CGC) for the optimized ratio of ADAMa25 was determined to be 0.6 w/v%. This CGC is not universal but is solely based on photopolymerization. The limiting factor for low‐concentration (≤0.6 w/v%) crosslinking of this composition is thus this covalent and irreversible methacryloyl‐bond. These characteristics are important for the optimization of ADAMa synthesis and development of low‐viscosity, low‐concentration hydrogels.

**FIGURE 4:**
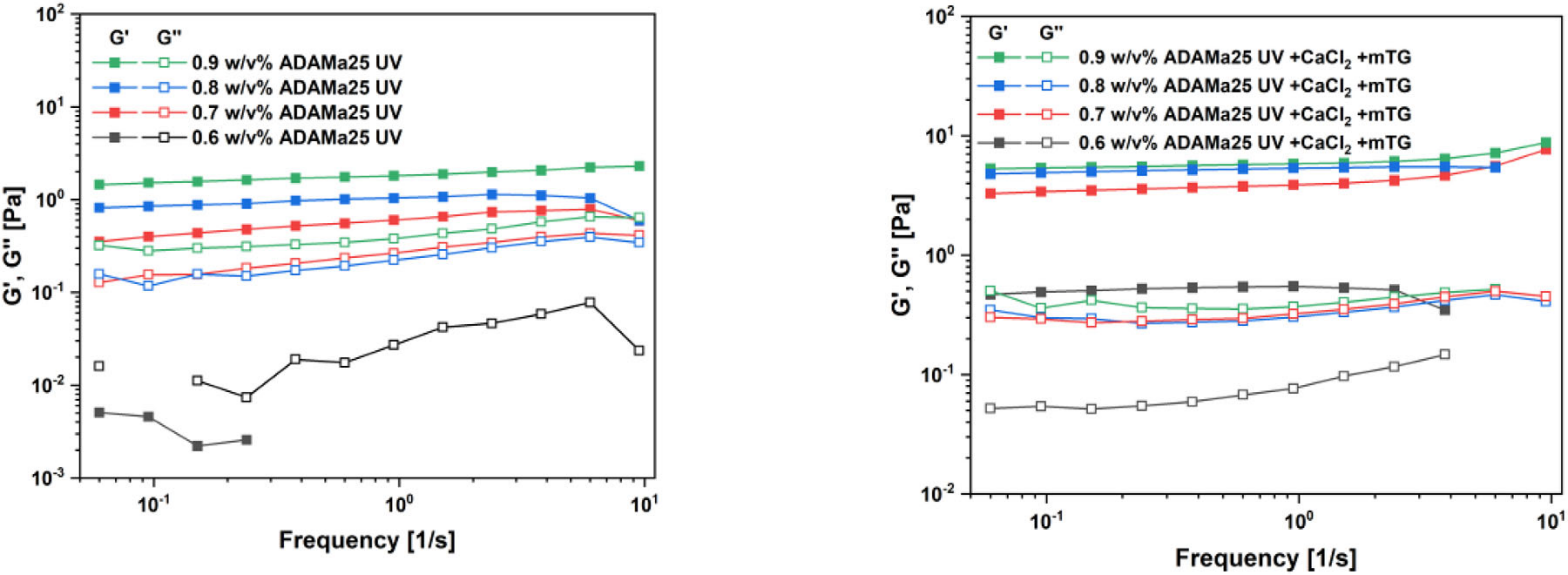
Storage and loss moduli for frequency sweeps of photocrosslinked (left) and multi‐crosslinked (right) ADAMa25 with different lyophilizate concentrations in 0.1 w/v% LAP and DPBS. Oscillation strain: 1%; testing temperature: 37°C; geometry: 40 mm plate‐plate.

Crosslinked hydrogel films, consisting of pure ADAMa, pure GelMa, and different mixing ratios, showed effective compressive moduli that ranged between 0.3 and 30 kPa (see Figure 5). An increase in GelMa content correlated with higher mechanical stiffness and an increase in hysteresis (see Figure S4). The lowest value was achieved for photocrosslinked ADAMa, while the highest value was measured for ADAMa25 after multi‐crosslinking. MAAh‐modified ADAMa with ionic and photocrosslinking was reported by another group to be around 15 kPa when compressed in air at 25°C and with a 0.05 s^−1^ strain rate [26]. Considering the higher strain rate and possible effects from testing in air, a higher effective modulus can be explained. Although different testing conditions, molecular weight, and photoinitiators were used, the measured values of 15 kPa to 12 ± 1 kPa are within the same order of magnitude. Effective compressive modulus values of 8 ± 3 kPa for 120 s photocrosslinked GelMa are within the right order of magnitude when compared with the literature, where concentrations, crosslinking, and testing conditions vary [42, 43, 44]. The dominating crosslinking mechanisms for GelMa and ADAMa have been reported before, and they can be tuned to varying degrees on their own [45]. The biggest contributors for each crosslinking mechanism of ADAMa‐GelMa are reflected in covalent photocrosslinking, as indicated by rheology, ionic crosslinking for ADAMa, and covalent crosslinking with transglutaminase for GelMa. It is important to note, however, that the ADAMa‐GelMa networks bond covalently, as indicated by a continuous phase in UV–vis spectrophotometry, forming a hybrid network, where the contribution and local distribution of the constituents may change the observed response [25]. In terms of measured compression modulus, however, the largest difference lies in the different elastic response of GelMa compared to the lower modulus ADAMa. Similarly, different compositions and concentrations are used to test ADA‐Gel; however, values from compression tests with both higher and lower strain rates range between 1–5 kPa, which is comparable to the values for higher ADAMa concentrations [43, 45, 46]. Multi‐crosslinked ADAMa‐GelMa films, on the other hand, starting from ADAMa50 where swelling was negligible, exhibit moduli of ≥20 kPa. The difference can be attributed to the irreversible covalent bonds from photocrosslinking. ADAMa100 showed noticeable swelling of up to more than 2.5 times its original volume compared to GelMa samples, where no swelling was found after storage for 1 day at 4°C (data not shown). This is assumed to be related to the low molecular weight and the hydrolytic degradation of ADAMa, hence also the low modulus [47].

**FIGURE 5:**
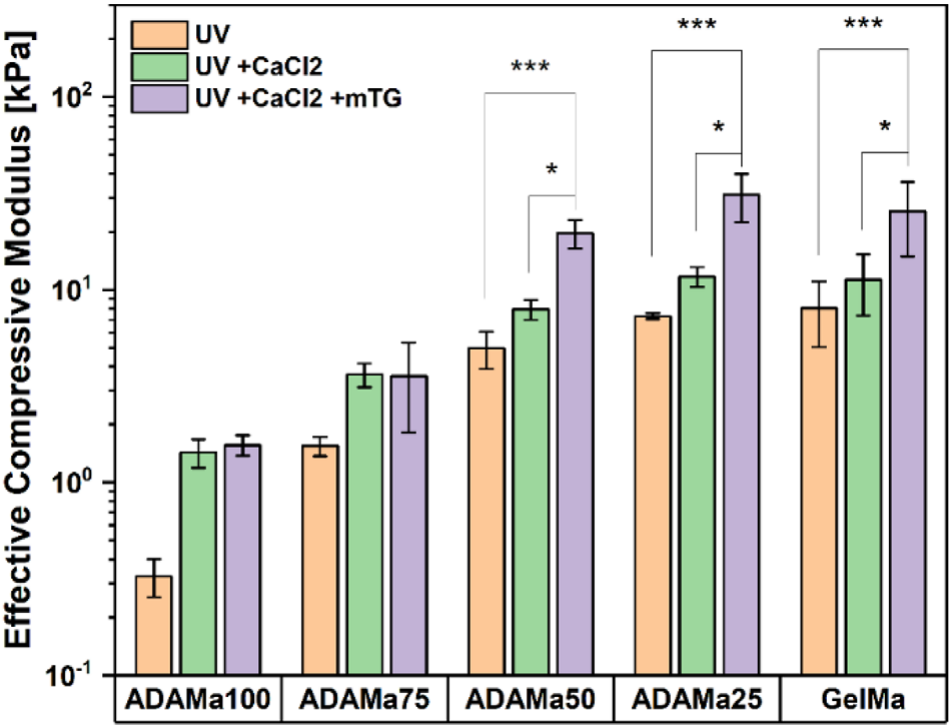
Effective compressive moduli measured at 0.005 s^−1^ strain rate in 37°C water bath for hydrogel films (n = 4) with 4 w/v% lyophilizate and 0.1 w/v% LAP in DPBS after cumulative crosslinking of ADAMa solution, GelMa solution and relative mixing ratios with 405 nm LED for 120 s and 0.1 M CaCl_2_ alone or with 2 w/v% mTG for 10 min and 1 day storage in DPBS at 4°C. Statistical significance was assessed with Tukey's Honestly Significant Difference using the following levels of significance: ^*^ *p* < 0.05, ^**^ *p* < 0.01, and ^***^ *p* < 0.001.

SEM of hydrogel cross‐sections shows an increase in porosity and irregular pore shapes and sizes for an ADAMa concentration ratio above 50% (see Figure S5). These pores are assumed to be related to degradation and could have affected the mechanical properties.

The development of bioinks for drop‐on‐demand bioprinting requires a careful balance between polymer molecular weight, viscosity, and post‐printing mechanical stability. While high‐molecular‐weight alginates provide excellent chain entanglement and mechanical strength, their elevated viscosity can limit droplet formation and printing reliability. Therefore, controlled molecular weight reduction can be advantageous by improving flow behavior while maintaining sufficient functional groups for subsequent crosslinking. In the present study, the selected alginate modification strategy was designed to optimize printability while enabling the formation of stable multi‐crosslinked hydrogel networks. Considering the results of mixing effects, gelation, and degradation, the resulting mechanical properties can be tailored over a broad range. This signifies the degree of versatility of this complex ADAMa‐GelMa system and points toward a large degree of freedom in the design of DoD bioprinted structures.

### 3.3 In Vitro Biocompatibility

For the in vitro evaluation, hydrogel samples were cast and subsequently seeded with human BJ1 fibroblasts. Figure 6 shows representative ADAMa25 and GelMa seeded surfaces with BJ1 hTERT cells spreading after 8 days. The cells demonstrate the characteristic elongated fibroblast morphology. Cell adhesion prior to solvent exchange and critical point drying appears to have been strong, as indicated by the close attachment of cells to the wavy dried material surface. Samples with higher ADAMa content show a more irregular, less comparable morphology and are thus not shown here (see Figures S5 and S6).

**FIGURE 6:**
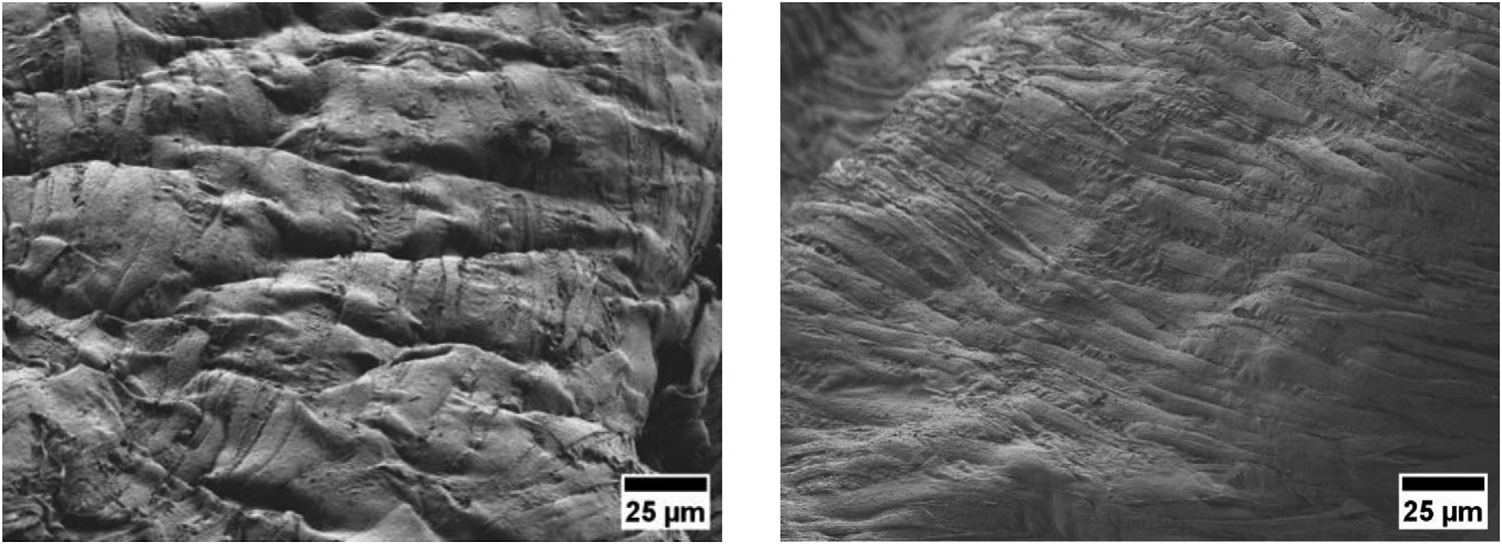
SEM images of critical‐point‐dried, ethanol‐infiltrated, glutaraldehyde‐ and formaldehyde‐fixed ADAMa25 (left) and GelMa (right) after crosslinking with 120 s irradiation at 405 nm and incubation in 0.1 M CaCl_2_ solution for 10 min. The samples were subsequently treated with additional mTG in CaCl_2_ solution and seeded with 10^5^ cells/mL BJ1 hTERT tdTomato fibroblasts on the hydrogel surfaces. All samples were incubated for 8 days in cell culture medium at 37°C and 5% CO_2_.

The maximum values for cell viability on ADAMa‐containing materials were obtained for the material with the highest GelMa content. This resulted in a maximum relative viability of approximately 270% for ADAMA25 compared with ADAMa100 after eight days (Figure 7). This outcome was anticipated and can be rationalised through the presence of RGD‐sequences in GelMa [48]. Despite the absence of a direct proportional relationship between the incorporation of GelMa and the observed outcomes, it is evident that there is no linear increase. It is hypothesised that the hydrolytically sensitive ester and low molecular weight are the causative factors in this phenomenon. Higher GelMa concentrations resulted in a denser cell layer (see also Figure S6) and more spread morphology (see Figure S7). Quantitatively, the evaluation of the WST‐8 assay shows a significant increase in cell viability within 8 days of incubation (see Figure 7). In the course of the in vitro biocompatibility tests, ADAMa100 had already undergone complete degradation, thus precluding the possibility of image capture. The present study demonstrates that the methacrylate reaction is the sole active irreversible crosslinking mechanism in this instance. ADAMa by itself lacks cell‐adhesive molecules but displays lower mechanical stiffness, different morphology, and high degradability (see Figure S5). The influence of LAP and radical formation, especially at such low concentrations at the used wavelength, was found to be negligible in preliminary studies and compares to other references in this field [26, 39, 49, 50].

**FIGURE 7:**
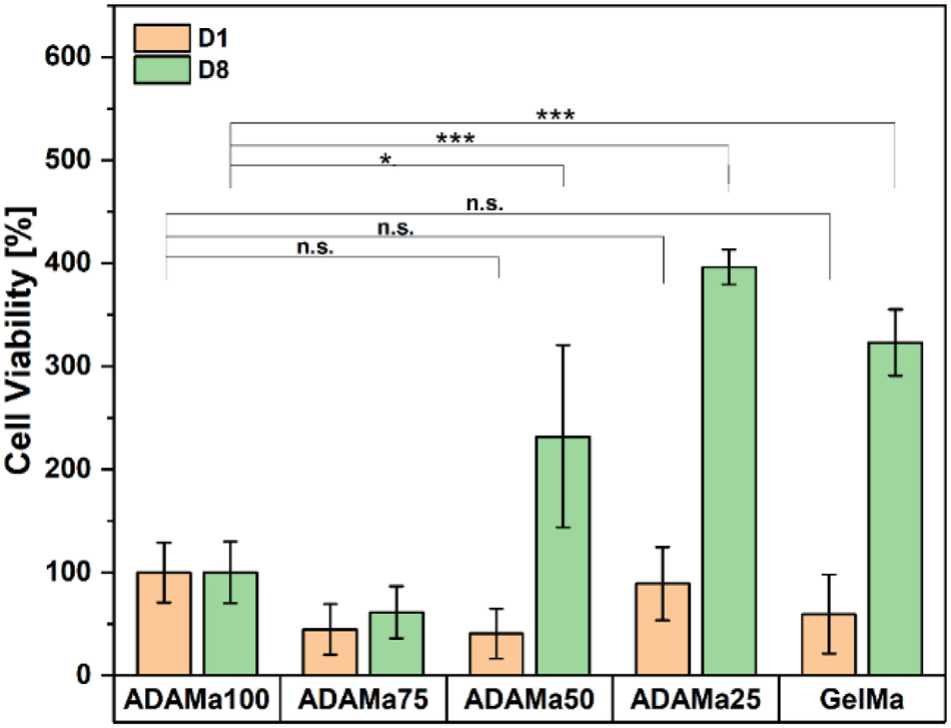
Evaluation of WST‐8 viability assay for BJ1 hTERT tdTomato fibroblasts seeded with 10^5 ^cell/mL concentration in cell medium on hydrogel films, containing 4 w/v% lyophilizate, 0.1 w/v% LAP and DPBS each after crosslinking with 405 nm for 120 s as well as 0.1 M CaCl_2_ and 2 w/v% mTG in a 96‐well plate and photometrically measured after 1 and 8 days of incubation at 37°C and 5% CO_2_. Statistical significance was assessed with Tukey's Honestly Significant Difference using the following levels of significance: ^*^ *p* < 0.05, ^**^ *p* < 0.01, and ^***^ *p* < 0.001.

In summary, the conducted in vitro tests do not demonstrate any indication of adverse interactions between the material and cells. Rather, they suggest a favorable outcome when combining ADAMa and GelMa, provided that the correct mixing ratio is utilised.

### 3.4 Bioprinting

#### 3.4.1 Printing Process Evaluation

These printing experiments were performed in the absence of cells, with the objective of analysing the relationship between bioink and the DoD printing process. Both FFT and GST show an increase in print quality with higher GelMa content; however, no significant differences in strut diameter or printability for different mixing ratios (see Figures 8 and 9). The mean overall diameter was 0.6±0.1 mm. The unevenness of struts with higher ADAMa concentrations may be related to low viscosity and clogging issues. An improvement in mechanistic printability by addition of ADAMa could not be shown in this study. Overall, the printability values were below 0.9. As such, the ink can be said to be undergelled [51]. FFT showed a printability down to 1 mm line distance. For GelMa, the printability was evaluated for a photocrosslinking duration of 30 and 120 s with GST (see Figure S8). The strut diameters showed no significant difference. Instead, significantly better printability (p = 0.015) and thus pore shape were observed for the exposure time of 120 s (see Figure S9). The increase in exposure time can be related to the crosslinking degree and viscosity, and thus, an enhancement in terms of shape stability. While the nozzle size could be increased to avoid clogging issues, these results indicate that the ADAMa ink still needs improvement, and GelMa alone is, in terms of printability, still the gold standard. However, the results also show that DoD printing is versatile enough to print low‐viscosity ink into structures, comparable to extruded filaments, without modifying parameters, such as strut size, by much.

**FIGURE 8:**
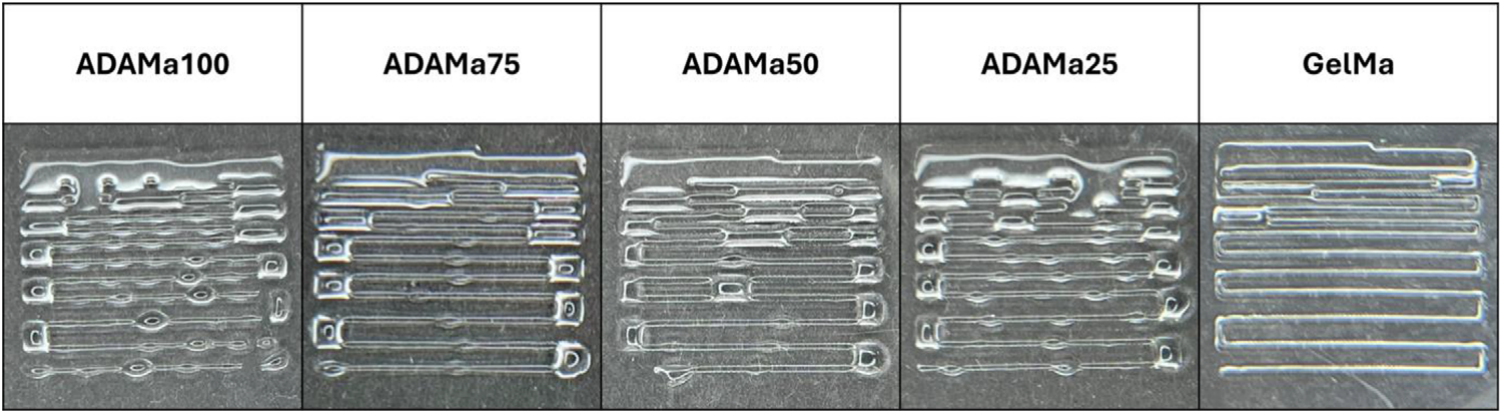
FFT by BioX Gen 3 printer for pure ADAMa and GelMa solutions as well as for different ADAMa‐GelMa mixing ratios at 4 w/v% lyophilizate concentration in DPBS with 0.1 w/v% LAP directly after printing at 37°C, 15 mm/s moving speed, with 1 ms opening and 25 ms closing time at printing pressure of 37 kPa for GelMa, 100 kPa for ADAMa25 and ADAMa50, 170 kPa for ADAMa75 and ADAMa100 and photocrosslinking for 30 s at 405 nm. Segments are shown with decreasing line distance from 2, 1.5, 1, 0.75 to 0.5 mm.

**FIGURE 9:**
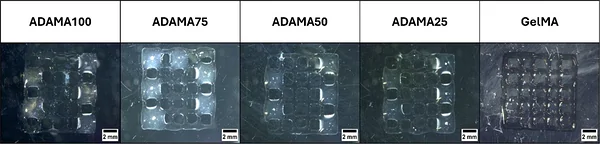
GST by BioX Gen 3 printer for pure ADAMa and GelMa solutions as well as for different ADAMa‐GelMa mixing ratios at 4 w/v% lyophilizate concentration in DPBS with 0.1 w/v% LAP directly after printing at 37°C,15 mm/s moving speed, with 1 ms opening and 25 ms closing time at printing pressure of 37 kPa for GelMa, 100 kPa for ADAMa25 and ADAMa50, 170 kPa for ADAMa75 and ADAMa100 and photocrosslinking both layers for 30 s at 405 nm each.

In a second step, 4 w/v% GelMa and ADAMa25 pillars of different sizes were printed vertically, layer‐wise. A maximal height of up to 2 mm was achieved for GelMa and was limited by the print head mobility in the z‐axis. For ADAMa25, a pillar height of 1 mm was achieved over 10 layers with 37°C cartridge temperature, 100 kPa pneumatic pressure, 15 mm/s print head speed, 1 ms valve opening and 100 ms valve closing time, and layer‐wise photocrosslinking at 405 nm for 30 s each (see Figure S10).

This was limited by clogging of the printhead after continuous printing, which may be related to sedimentation of particles when printing low‐viscosity inks for prolonged time [52]. The first layer of hydrogel pillars was relatively spread and thus discounted from the layer count. The diameter in the middle part of the pillar was set to 0.5 mm for this particular configuration. Vertical structures with this aspect ratio and a layer count of 10 for ADAMa‐GelMa are unique and promise an adequate feature size for a range of applications in biomedical research [2, 12].

The fabrication of hydrogel pillars was achieved through a printing process with a sufficient distance maintained between each individual pillar (refer to Figure S11). The volume of the resulting hydrogel was approximately 1.5 µL, derived from four individual drops, each with a volume of 500 nL. A minimal line distance, larger than 1 mm, was determined for 2 mm‐high GelMa pillars to stand upright without fusing. This means that the previously determined limit for FFT of 1 mm does not always translate to higher layer counts. ADAMa25 was stable without fusion of pillars up to a layer count of 7 and a line distance of 2 mm (see Figure 10). Further tests could be performed; however, the stability is expected to be lower than for GelMa pillars, considering the lower compressive modulus. Environmental, material, and printing parameters are all expected to affect these results. With this, an initial three‐dimensional printability test to determine three‐dimensional resolutions is proposed for DoD bioprinting. Further tests regarding mechanical properties, degradation behavior, and droplet interfaces in hydrogel pillars are within the scope of future testing.

**FIGURE 10:**
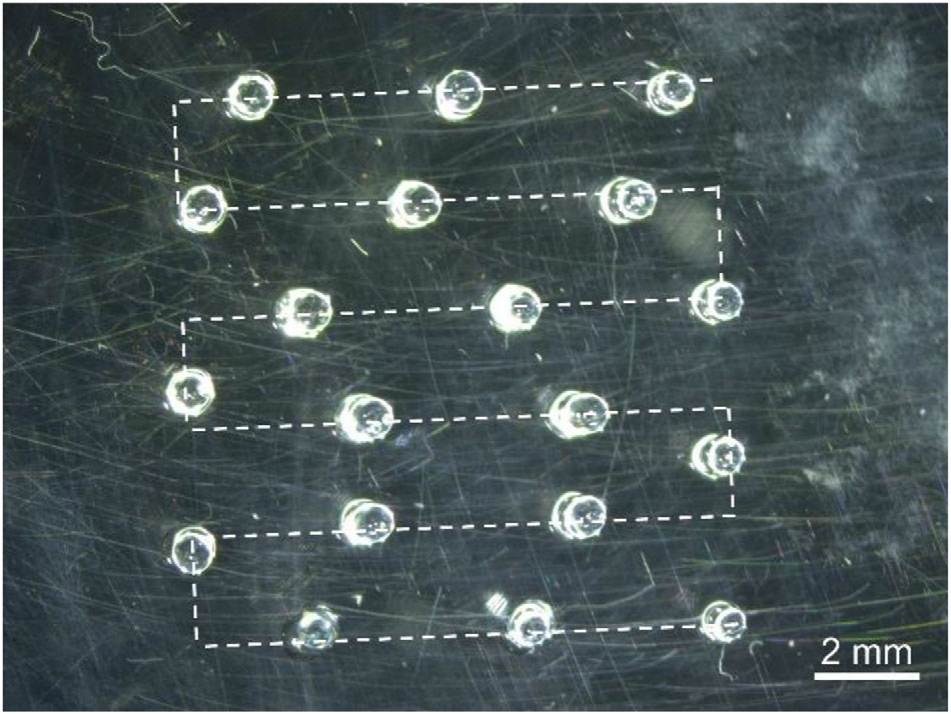
4 w/v% ADAMa25 pillar with 6 layers and line distance of 2 mm by BioX Gen 3 printer directly after printing at 37°C, 15 mm/s moving speed, with 3 ms opening and 200 ms closing time and at printing pressure of 100 kPa.

#### 3.4.2 Printing with Cells

Three‐dimensional millimeter‐sized hydrogel cell‐laden structures were printed with both GelMa and ADAMa‐GelMa (see Figure S12). The distribution of cells was quantified using multiphoton microscopy analysis on 4 wt% GelMa with 1 million cells per milliliter (see Figure 11). When considering the outward force upon impact and the diameter of the pillar, which is up to around 500 µm for 4 w/v% GelMa with the used parameters, compared to the layer height of around 100 µm, the difference in closest cell‐cell distance can be explained. In the multiphoton image analysis of the distribution for the vertical direction of GelMa pillars, the majority of closest cell‐cell distances are below this characteristic value of layer height. No effect from the layers and weld lines was visible. On the other hand, the horizontal distribution showed a larger distribution (see Figure S13). The median values for the print in the horizontal and vertical directions are 56 ± 34 µm and 45 ± 31 µm, respectively, and are comparable to reference values of 67 ± 4 µm [53]. This showcases the bioprintability of this system and can act as a reference for further studies, where the system and parameters may be further optimized. Interestingly, cell aggregation could also arise from the specific conditions associated with DoD bioprinting. Xu et al. demonstrated that both bioink concentration and printing duration strongly influence cell aggregation during DoD processing [52]. Therefore, aggregation in cell‐laden droplets is likely governed by a complex interplay between polymer concentration, viscosity, printing parameters, and cell–cell interactions. These findings highlight the importance of optimizing both bioink formulation and printing strategy for achieving application‐specific cellular organization.

**FIGURE 11:**
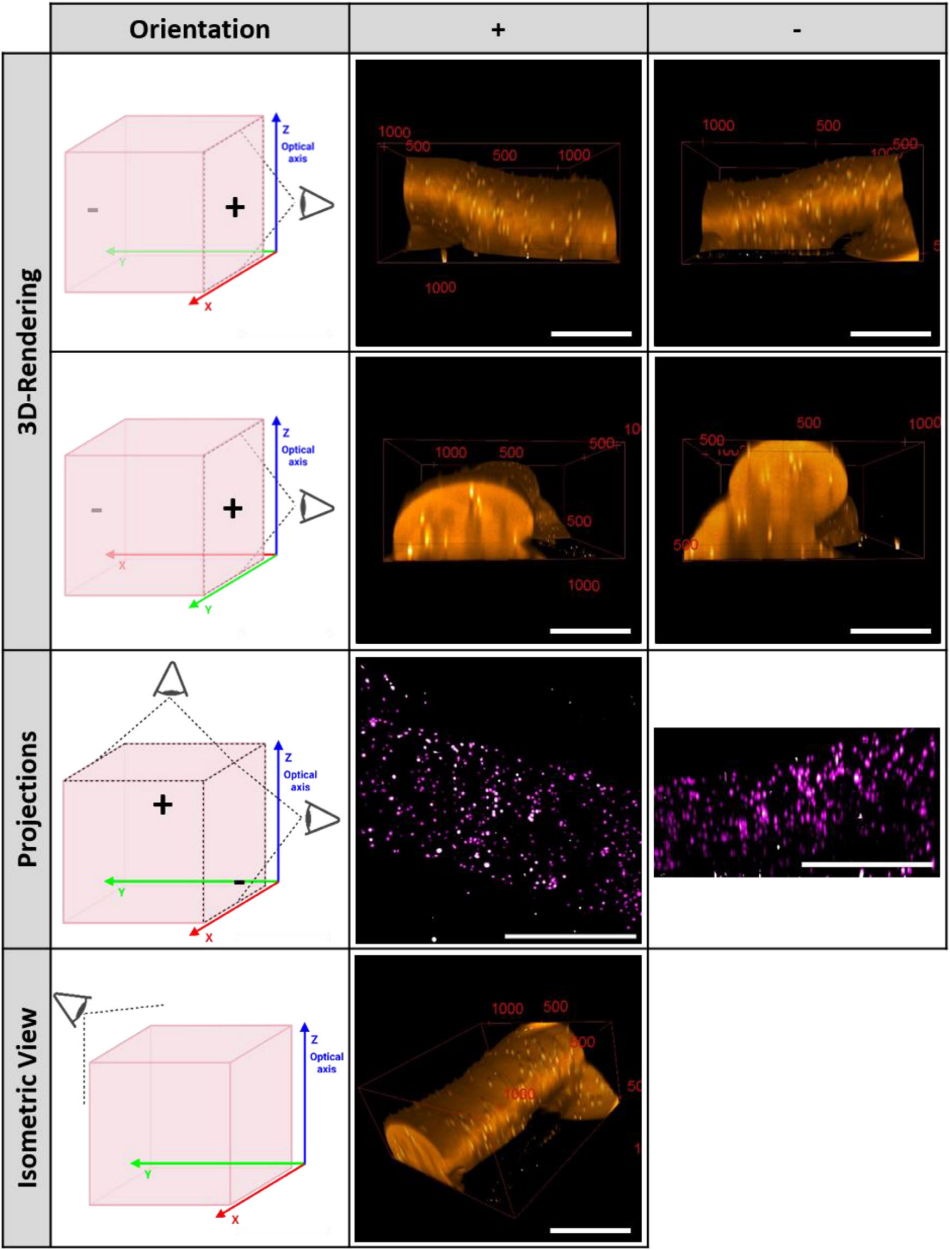
Analysis of multiphoton microscopy of 3D rendered multiphoton images of the pillar‐shaped bioprinted construct, as well as Z‐Projections of the respective 3D image stacks, to show an even distribution of cells throughout the construct. The coordinate systems in the first column depict the exact point‐of‐view of the observer in correlation to the extrusion axis of the 3D printer (Z). The isometric view in the lowest row provides additional insight into the three‐dimensional structure of the printed structures. The scale bar is 500 µm.

Similar distances to MPM are seen for ADAMa25 with SEM surface images and light microscopy bulk images of pillars (see Figure 12A–D). SEM image shows that encapsulated cells were distributed and remained along the surface of 4 w/v% ADAMa25 after crosslinking at 405 nm for 30 s per layer, 0.1 M CaCl_2_ for 10 min, and freeze‐drying of ethanol‐infiltrated samples. This demonstrates the homogeneous distribution of cells after printing and the printability of pillars up to 2 mm in height using DoD bioprinting.

**FIGURE 12:**
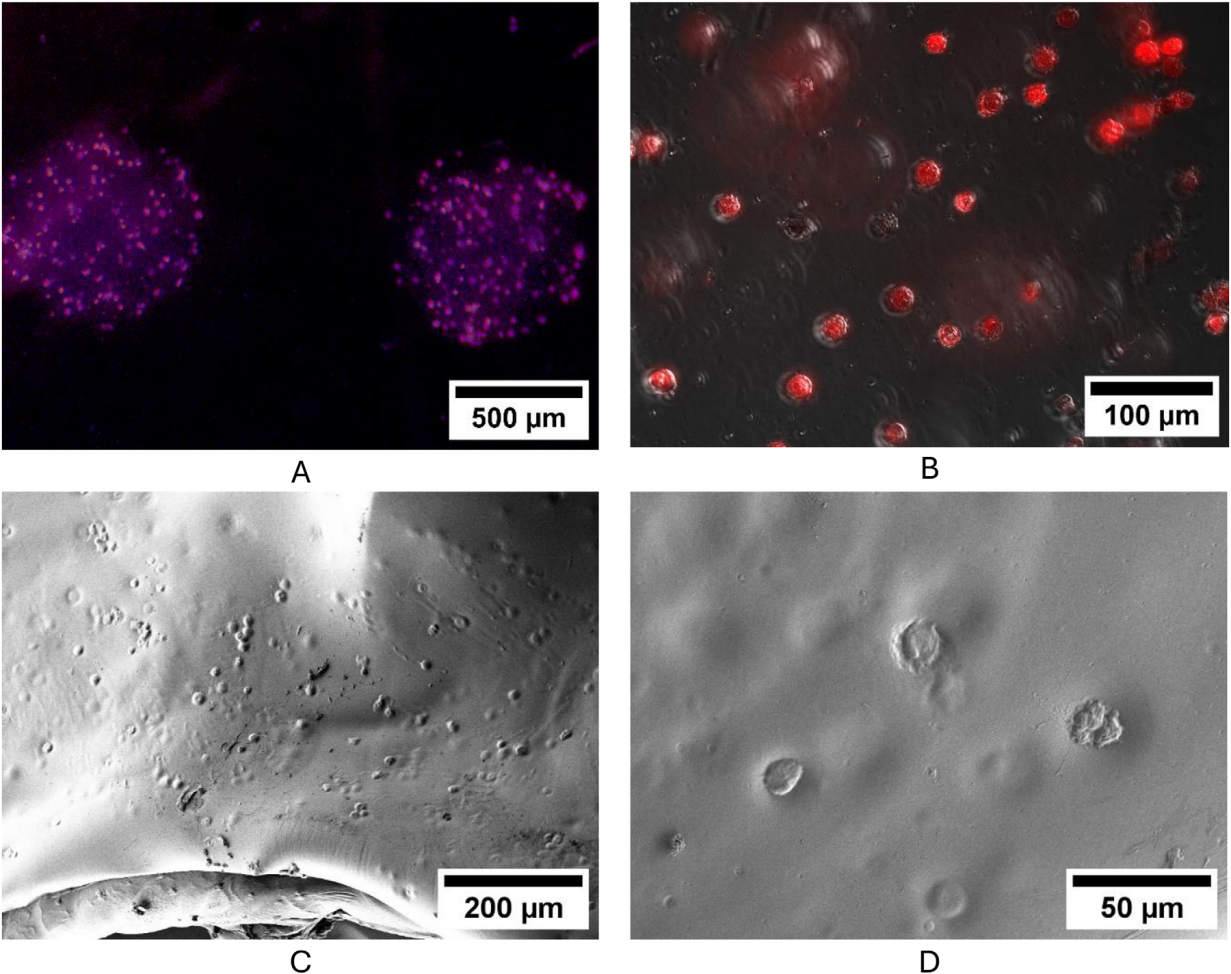
A: Light microscopy of 3‐layer pillars in DMEM and at higher magnification with overlaid fluorescence microscopy (B) after 1 day of incubation at 37°C and 5% CO_2_ for BJ1 hTERT tdTomato fibroblasts at a concentration of 10^6 ^cell/mL encapsulated within 4 w/v% ADAMa25 0.1 w/v% LAP and DPBS after crosslinking with 405 nm for 30 s for each of 3 layers and 0.1 M CaCl_2_ for 10 min, printed with 37°C cartridge temperature,100 kPa pressure, 3 ms opening and 100 ms closing time at 15 mm/s print head speed. C‐D: SEM pictures at different magnification levels of BJ1 fibroblasts with a concentration of 10^6 ^cell/mL at the surface of 4 w/v% ADAMa25 0.1 w/v% LAP and DPBS after printing at 37°C, 15 mm/s moving speed, with 3 ms opening and 200 ms closing time, at printing pressure of 100 kPa, crosslinking with 405 nm for 30 s for each layer of a 3‐layer pillar.

For GelMa and ADAMa25 pillars printed with encapsulated cells, a diameter for the first layer of around 0.9 mm and 1.1 mm was found (see Figure S12). This can be attributed to material properties, printing parameters, substrate shape, stiffness, and interaction parameters.

The majority of the embedded BJ1 cells appear to be round in shape. Following a 24 h period of incubation in cell medium, evidence of cell elongation in printed GelMa structures became apparent (see Figure S12). This finding cannot be directly compared to the morphology of seeded cells and may be influenced by factors such as cell adhesion, hydrogel density, radical activity in the printed structure, or high shear rates during printing. Nevertheless, visible cell elongation after 24 h of incubation is a promising initial result for a high hydrogel concentration of 4 w/v% GelMa. A number of alternative approaches to enhance cell spreading inside the hydrogel are conceivable. These include the incorporation of additional cell‐adhesive molecules, a reduction in polymer concentration, the utilisation of non‐radical photopolymerisation, the incorporation of protective additives such as plasticisers for printing with cells, or an increase in nozzle size to reduce shear stress [54, 55].

## 4 Conclusion

Here, we developed a multi‐crosslinkable ADAMa‐GelMa bioink specifically designed for drop‐on‐demand bioprinting. The combination of methacrylated oxidized alginate and GelMa enabled the generation of a versatile hydrogel platform that integrates the structural advantages of alginate with the biofunctional and mechanical contributions of gelatin. The controlled modification of alginate shows a suitable balance between polymer functionality and rheological properties required for reliable droplet‐based printing. The resulting ADAMa‐GelMa system exhibited tunable mechanical properties through the synergistic combination of photopolymerization, dynamic covalent interactions, and ionic crosslinking mechanisms. This multi‐crosslinking strategy enabled the adjustment of hydrogel properties while maintaining the low‐viscosity characteristics essential for DoD processing. The developed bioink demonstrated good printability, cytocompatibility, and the ability to generate three‐dimensional cell‐containing hydrogel structures with defined spatial organization. Among the investigated formulations, ADAMa25 showed particularly favourable characteristics by combining reproducible printing performance with suitable mechanical and biological properties. Overall, this study establishes ADAMa‐GelMa as a flexible bioink platform for DoD bioprinting and provides a foundation for the development of application‐specific hydrogel constructs in advanced biofabrication.

## Author Contributions

**Tommi Bui**: investigation, methodology, software, Writing – original draft. **Emine Karakaya**: writing – original draft, conceptualization, formal analysis. **Jessica Faber**: writing – original draft, methodology. **Jonas Röder**: validation, software. **Jörg Teßmar**: investigation, validation, formal analysis. **Julian Bauer**: investigation, validation, formal analysis. **Oliver Friedrich**: writing – review and editing, formal analysis, validation. **Silvia Budday**: writing – review and editing, resources, validation. **Aldo R. Boccaccini**: writing – review and editing, formal analysis, supervision. **Rainer Detsch**: conceptualization, writing – original draft, writing – review and editing, project administration, supervision.

## Conflicts of Interest

The authors declare no conflicts of interest.

## Supporting information




**Supporting File**: mabi70278‐sup‐0001‐SuppMat.docx.

## Data Availability

The data that support the findings of this study are available on request from the corresponding author. The data are not publicly available due to privacy or ethical restrictions.
