# Supplementary material for: A Multi‐Crosslinkable Methacrylated Alginate‐GelMa Bioink for Drop‐on‐Demand Bioprinting
Source: Macromol Biosci. 2026 Oct 31;26(11):e70278. doi: 10.1002/mabi.70278 (PMC13723047; doi:10.1002/mabi.70278)
Supplement: Supplementary file 1 [file MABI-26-e70278-s001.docx]

**Supplements:**

**A Multi-Crosslinkable Methacrylated Alginate-GelMa Bioink for Drop-on-Demand Bioprinting**

Tommi Bui^1*^, Emine Karakaya^1*^, Jessica Faber^2^, Jonas Röder^1^, Jörg Teßmar^3^, Julian Bauer^4^, Oliver Friedrich^4^, Silvia Budday^2^, Aldo R. Boccaccini^1^ & Rainer Detsch^1#^

^1^Institute of Biomaterials, Department of Materials Science and Engineering,

Friedrich-Alexander-University Erlangen-Nürnberg, 91058 Erlangen, Germany

^2^Institute of Continuum Mechanics and Biomechanics, Department of Mechanical Engineering,

Friedrich-Alexander-University Erlangen-Nürnberg, 90762 Fürth, Germany.

^3^Department for Functional Materials in Medicine and Dentistry, University of

Würzburg, Germany

^4^Institute of Medical Biotechnology, Department of Chemical and Biological

Engineering, Friedrich-Alexander-University Erlangen-Nürnberg, 91056 Erlangen,

Germany

*These authors contributed equally to this work.

\# Corresponding author: [rainer.detsch@fau.de](mailto:rainer.detsch@fau.de)

**Chemical analysis**

NMR measurements:

Table S1: Regions and values of all parameter used for the determination of %methylacrylation.

| **Chemical shift (ppm)** | **Integral (I)** | **Assignment** | **Number of protons (n)** | **Normalized integral (I_N_)** | **I_N_ / n** | **% meth-acrylation** |
| --- | --- | --- | --- | --- | --- | --- |
| 6.8-6.1 ppm | 2.85 | Vinyl | 2 | 0.4867 | 0.2433 | ~ 24.3 % |
| 2.4-2.2 ppm | 3.15 | Methyl | 3 | 0.5380 | 0.1793 | ~ 17.9 % |
| 5.7-3.9 ppm | 112.01 | Backbone + HDO | 4 + x |  |  |  |
| 4.8-4.6 ppm | 88.59 | HDO | x |  |  |  |
|  | 23.42 | Backbone | 4 | 4 | 1 |  |

ATR-FTIR Spectroscopy:

ATR-FTIR spectra (IRAffinity-1S, Shimadzu, Japan) in Fig. S1 show characteristic peaks for Alg, ADA and their methacrylic anhydride based derivates. In pre-tests, AlMa was synthesized similar to ADAMa. Inconsistent ATR-FTIR spectral signal ratios despite using the same methods and material, probably due to inconsistent material thickness, surface contact and possible raw material heterogeneity among other things, made it difficult to quantify the degree of functionalization reliably with this method. NMR was used for quantification instead.
When comparing reference spectra of Alg and ADA (13% oxidation) compared to their methacrylates in ATR-FTIR spectroscopy, an additional carbonyl stretching band at 1720 cm^-1^ is found after functionalization is attributed to the formation of methacrylate ester group with otherwise no indication of side products.

Fig. S1: ATR-FTIR spectra for alginate (Alg) powder and freeze-dried alginate-dihaldehyde (ADA)as well as freeze-dried MAAh modified Alginate (ALMa) and ADAMa within 4000-400 cm^-1^ with scan count of 40 and . 4 cm^-1^ resolution

**Physical Characterization and Mechanical Analysis**

UV-Vis spectroscopy:

UV-Vis spectroscopy was conducted with Specord 40 spectrophotometer (Analytik Jena AG, Germany) using DPBS as background to record the absorption of the precursor solutions within the range from 320 to 1100 nm (see Fig. S2). LAP showed a consistent absorption peak at around 375 nm and a moderate absorption level at 405 nm. Baseline shift for ADAMa75 and ADAMa50 is attributed to scattering at liquid-liquid interfaces.

Fig. S2: UV-Vis absorbance spectra for pure DPBS as background for 0.1 w/v% LAP as reference and additional 1 w/v% lyophilizate dissolved in DPBS for ADAMa, GelMa and mixed ADAMa-GelMa precursors

Rheology:

Thermal gelation of 0.6 w/v% ADAMa25 was characterized using a parallel plate rheometer (HR30, TA Instruments, USA) equipped with a stainless steel plate-plate geometry (Plate SST, diameter of 40 mm) and a gap of 500 μm (see Fig. S3). The peltier cooling system was used to set a cooling rate of 1 °K/min, decreasing the temperature from 20°C to 8°C. The frequency for this temperature ramp measurement was 10 rad/s. Crossover point for these conditions is around 0.035 Pa at 14 °C.

Fig. S3: Storage and loss moduli for a temperature ramp measured for 0.6 w/v% ADAMa25 precursor with 0.1 w/v% LAP and DPBS and no further crosslinking

Mechanical tests:

Stress-strain curves were measured by Microtester LT (CellScale, Canada) at 37 °C in a water bath with 1 pre-cycle and 3 measurement cycles. Each cycle deformed the sample to a maximum strain of 10% within 20 s in an initial step, held the position for 5 s, released the force during 20 s and then waited for 5 s for the sample to recover. A linear range was defined for 2-5% strain (see Fig. S4).

Fig. S4: Representative stress-strain curves measured at 37 °C of hydrogel films with 4 w/v% lyophilizate and 0.1 w/v% LAP in DPBS after photocrosslinking of ADAMa solution, GelMa solution and relative mixing ratios at 405 nm for 120 s and 1 night of storage in DPBS at 4 °C.

Microscopy:

SEM (Auriga CrossBeam Unit, Carl Zeiss, Germany) was done after crosslinking films with 0.1% glutaraldehyde and 2% paraformaldehyde for overnight and then for 1 h with 0.3% glutaraldehyde and 3% paraformaldehyde. The samples were critical point dried (EP CPD300, Leica, Germany) once infiltrated with ethanol solutions (30, 50, 70, 80, 90, 95, 99.5%) for 25 min each in corresponding order.

The hydrogel cross-sections show an increase in porosity, irregular pore shapes and sizes for an ADAMa concentration ratio above 50% (see Fig. S5). These pores are assumed to be related to degradation and affect the mechanical properties.

Fig. S5: SEM images of cross-sections for critical point dried ethanol infiltrated glutaraldehyde and formaldehyde fixed 4 w/v% lyophilizate hydrogel with 0.1 w/v% LAP in DPBS after crosslinking with 405 nm for 120 s and 0.1 M CaCl_2_ for 10 min.

***In Vitro* Biocompatibility**

Fluorescence microscopy and SEM images were taken for human BJ1 hTERT tdTomato fibroblasts, seeded and incubated on ADAMa-GelMa films and prepared as above described after an incubation time of 8 days at 37 °C and 5 % CO_2_ (see Fig. S6). The fibroblastic phenotype is clearly expressed on all the samples. The images show the fibroblasts spreading and attaching with increasing GelMa content, at higher magnification pure GelMa, the elongation of BJ1 fibroblasts is particularly pronounced. (see Fig. S7).

Fig. S6: Overlay of light and fluorescence microscopy (top row) and SEM images of fixed samples (bottom row) after 8 days of incubation. The samples were seeded with 105 cell/mL BJ1 hTERT tdTomato fibroblasts in cell medium seeded on hydrogel films consisting of 4 w/v% lyophilizate and 0.1 w/v% LAP in DPBS after crosslinking with 405 nm for 120 s as well as 0.1 M CaCl2 and 2 w/v% mTG in a 96-well plate as single polymer phase or with relative mixing ratios except for pure ADAMa due to complete degradation.

Fig. S7: SEM image with high magnification of BJ1 hTERT tdTomato fibroblasts on 4 w/v% GelMa surfaces after 8 days of cultivation.

**Bioprinting**

Printability tests:

Grid Structure Test (GST) was performed for 4 w/v% GelMa (see Fig. S8). Crosslinking was done after each of the two layer prints. Printing parameters were 37 °C for temperature, 37 kPa for pressure, 15 mm/s for print head speed, and1 ms for valve opening and 25 ms for closing time of BioX printer (Gen 3, CELLINK, USA). The UV exposure was set at 405 nm, 6 cm height and 100% power for 30 and 120 s each. The printability was calculated from the measured circularity (*(Fiji Is Just) ImageJ* software (Version 2.9.0/1.53t)):

$$Printability= \frac{\pi}{4}\cdot\frac{1}{Circularity}$$

and then evaluated (see Fig. S9).

Fig. S8: GST with BioX Gen 3 printer, 37 °C cartridge temperature, 37 kPa pneumatic pressure, 15 mm/s print head speed, 1 ms valve opening and 25 ms valve closing time and layer-wise 405 nm photocrosslinking for 4 w/v% GelMa with 0.1 w/v% LAP in DPBS at different irradiation times

Fig. S9: Printability evaluation (n=4) from GST with BioX Gen 3 printer, 37 °C cartridge temperature, 37 kPa pneumatic pressure, 15 mm/s print head speed, 1 ms valve opening and 25 ms valve closing time and layer-wise 30 s or 120 s photocrosslinking at 405 nm for 4 w/v% GelMa with 0.1 w/v% LAP in DPBS at different irradiation times

ADAMa25 pillar shows around 1 mm in height and 0.5 mm in diameter (see Fig. S10).

Fig. S10: Sideway picture of 11-layer ADAMa25 directly after print with BioX Gen 3 printer, 37 °C cartridge temperature, 100 kPa pneumatic pressure, 15 mm/s print head speed, 1 ms valve opening and 100 ms valve closing time and layer-wise photocrosslinking at 405 nm for 30 s each

Filament fusion test for consistent line distances per tested layer height shows a resolution limit for 20-layer 4 w/v% GelMA with 0.1 w/v% LAP in DPBS at above 1 mm (see Fig. S11).

|  |  |
| --- | --- |
| Fig. S11: Three-dimensional fusion test of 4 w/v% GelMa pillars printed by BioX Gen 3 printer at 37 °C cartridge temperature, 37 kPa pressure, with 15 mm/s print head speed, 1 ms valve opening and 200 ms valve closing time, layer-wise 30 s photocrosslinking at 405 nm and increasing layer height of 0.1 mm with line distance of 5 mm (left) and 1 mm (right) | |

Microscopy:

The shape of GelMa and ADAMa pillars with encapsulated BJ1 hTERT tdTomato fibroblasts is shown in Fig. S12. ADAMa pillar with encapsulated cells showed a conical shape due to a clogged printing nozzle, which may have led to smaller droplets being ejected with increasing layer count. On the other hand, at the tip of samples, GelMa was found to show a hole, which is assumed to be caused by insufficiently crosslinked material, that is washed away during liquid post-crosslinking). The uppermost layer is photocrosslinked for 30 s whereas the lower layers are exposed to transmitted radiation for extended periods of time. Thus, sufficiently long photocrosslinking may have been missing and is necessary for structural integrity before contact-based liquid crosslinking methods can be used. The higher magnified picture in the bottom-left corner shows some fibroblasts expanding after 1 day of incubation in cell medium. The rest of the cells seem rather round.

|  |  |
| --- | --- |
|  |  |
| Fig. S12: Overlaid light and fluorescence microscopy images after 1 day of incubation for 20-layer 4 w/v% GelMa hydrogel pillars with different magnification levels (top-left, top-right, bottom-left) and of 5-layer 4 w/v% ADAMa25 (bottom-right) in DPBS with 0.1 w/v% LAP after photocrosslinking for 30 s at 405 nm and moving the printhead vertically by 0.1 mm after every layer print | |

The closest cell-cell distribution was recorded for the GelMa pillar with encapsulated cells. The distribution was skewed, depending on the direction of measurement.

|  |  |
| --- | --- |
| Fig. S13: Cell distribution of BJ1 hTERT tdTomato fibroblasts with a concentration of 10^5^ cell/mL (left) in pillars of 4 w/v% GelMa, 0.1 w/v% LAP and DPBS by measuring individual cell-cell distances within slices of multiphoton images (right) of a 21-layer pillar lying and a 3-layer pillar standing | |
